# Supplementary material for: Growth Optimisation and Kinetic Profiling of Diesel Biodegradation by a Cold-Adapted Microbial Consortium Isolated from Trinity Peninsula, Antarctica
Source: Biology (Basel). 2021 Jun 2;10(6):493. doi: 10.3390/biology10060493 (PMC8228002; doi:10.3390/biology10060493)
Supplement: Supplementary file 1 [file biology-10-00493-s001.zip › biology-1143065-SI.pdf]

Supplementary data

S1: One-way analysis of variance for primary screening of 28 soil consortia

one-way ANOVA and post-hoc Dunnett test	Mean Difference	q	p value	Significance
CONTROL vs BS1	-32.206	8.085	p<0.01	**
CONTROL vs BS2	-39.973	10.035	p<0.01	**
CONTROL vs BS3	-39.682	9.962	p<0.01	**
CONTROL vs BS4	-48.614	12.205	p<0.01	**
CONTROL vs BS5	-47.837	12.01	p<0.01	**
CONTROL vs BS6	-27.546	6.915	p<0.01	**
CONTROL vs BS7	-56.866	14.276	p<0.01	**
CONTROL vs BS8	-35.604	8.938	p<0.01	**
CONTROL vs BS9	-66.866	16.787	p<0.001	***
CONTROL vs BS10	-20.168	5.063	p<0.01	**
CONTROL vs BS11	-43.663	10.962	p<0.01	**
CONTROL vs BS12	-41.332	10.377	p<0.01	**
CONTROL vs BS13	-42.983	10.791	p<0.01	**
CONTROL vs BS14	-13.177	3.308	p<0.05	*
CONTROL vs BS15a	-64.731	16.251	p<0.001	***
CONTROL vs BS15b	-69.973	17.567	p<0.001	***
CONTROL vs BS16	-12.303	3.089	p<0.05	*
CONTROL vs BS17	-2.982	0.7486	p>0.05	ns
CONTROL vs BS19	-19.488	4.892	p<0.01	**
CONTROL vs BS20	-47.546	11.936	p<0.01	**
CONTROL vs BS21	-16.851	4.23	p<0.01	**
CONTROL vs BS22	-47.352	11.888	p<0.01	**
CONTROL vs BS23	-73.08	18.347	p<0.001	***
CONTROL vs BS24	-74.926	18.81	p<0.001	***
CONTROL vs BS25	-13.274	3.332	p<0.05	*
CONTROL vs BS26	-73.954	18.313	p<0.001	***
CONTROL vs BS27	-58.905	14.586	p<0.01	**
CONTROL vs BS28	-56.478	13.985	p<0.01	**

One-way ANOVA with post-hoc Dunnett's test (group vs control) implemented to shortlist the consortium with diesel biodegradation potential. Only samples with $p<0.001$ were selected for secondary screening at 2% v/v diesel concentration.

S2: One-way analysis of variance for secondary screening of six shortlisted soil consortia

TPH degradation (%)				
one-way ANOVA and post-hoc Tukey's test	Mean Difference	q	p value	Significance
BS9 vs BS15a	5.236	3.368	P>0.05	ns
BS9 vs BS15b	21.278	13.688	P<0.001	***
BS9 vs BS23	-0.336	0.2162	P>0.05	ns
BS9 vs BS24	-3.170	2.039	P>0.05	ns
BS9 vs BS26	29.107	18.725	P<0.001	***
BS15a vs BS15b	16.042	10.32	P<0.001	***
BS15a vs BS23	-5.572	3.584	P>0.05	ns
BS15a vs BS24	-8.405	5.407	P<0.05	*
BS15a vs BS26	23.871	15.357	P<0.001	***
BS15b vs BS23	-21.614	13.905	P<0.001	***
BS15b vs BS24	-24.448	15.728	P<0.001	***
BS15b vs BS26	7.829	5.036	P<0.05	*
BS23 vs BS24	-2.834	1.823	P>0.05	ns
BS23 vs BS26	29.443	18.941	P<0.001	***
BS24 vs BS26	32.277	20.764	P<0.001	***

*p <0.05, ** p < 0.01, *** p < 0.001.

Microbial growth ($OD_{600\ nm}$)				
one-way ANOVA and post-hoc Tukey's test	Mean Difference	q	p value	Significance
BS9 vs BS15a	0.2820	35.184	P<0.001	***
BS9 vs BS15b	0.4217	52.609	P<0.001	***
BS9 vs BS23	0.1823	22.749	P<0.001	***
BS9 vs BS24	-0.2797	34.893	P<0.001	***
BS9 vs BS26	0.4903	61.177	P<0.001	***
BS15a vs BS15b	0.1397	17.426	P<0.001	***
BS15a vs BS23	-0.0997	12.435	P<0.001	***
BS15a vs BS24	-0.5617	70.077	P<0.001	***
BS15a vs BS26	0.2083	25.993	P<0.001	***
BS15b vs BS23	-0.2393	29.861	P<0.001	***
BS15b vs BS24	-0.7013	87.502	P<0.001	***
BS15b vs BS26	0.0687	8.567	P<0.001	***
BS23 vs BS24	-0.4620	57.642	P<0.001	***
BS23 vs BS26	0.3080	38.428	P<0.001	***
BS24 vs BS26	0.7700	96.069	P<0.001	***

*p <0.05, ** p < 0.01, *** p < 0.001.

BS24 performed slightly better than the others in terms of microbial growth as verified through one-way ANOVA with post-hoc Tukey's test (multiple groups comparison). The authors proceed with BS24 although BS9, BS15a and BS23 were similarly weighted in terms of TPH degradation.

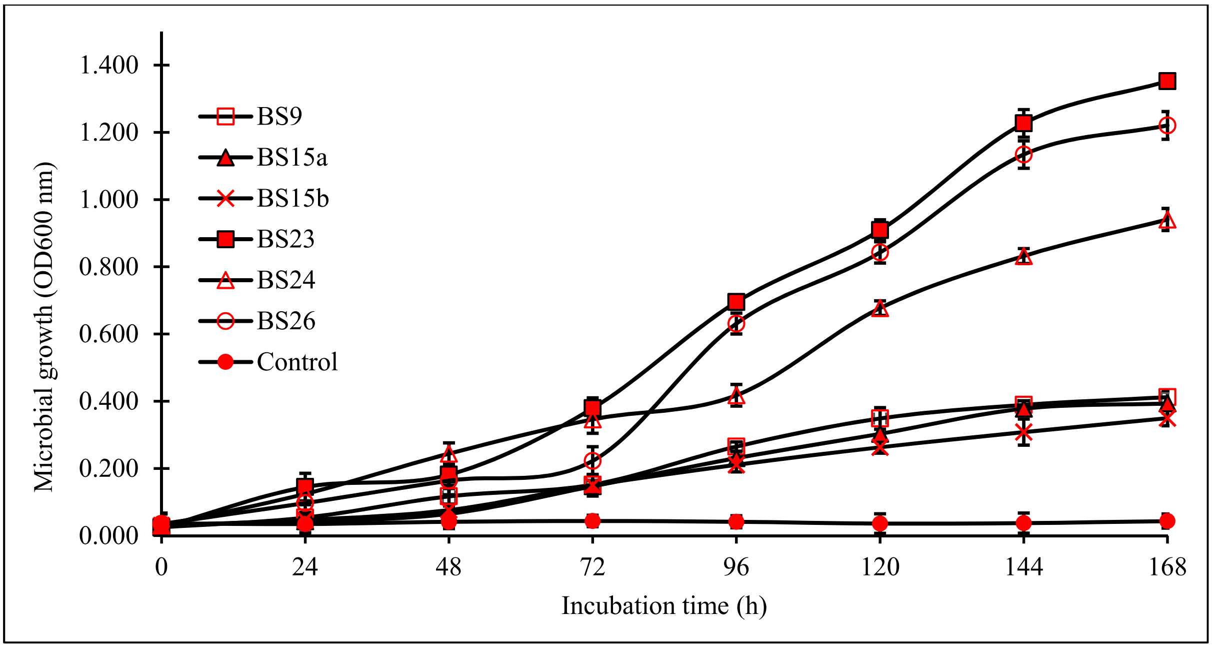

S3: The growth curves of six selected soil consortia over 7 d incubation time. Error bars represent the mean ± standard deviation for experimental triplicates.
